# Constraining the atmospheric OCS budget from sulfur isotopes

**DOI:** 10.1073/pnas.2007260117

**Published:** 2020-08-05

**Authors:** Shohei Hattori, Kazuki Kamezaki, Naohiro Yoshida

**Affiliations:** ^a^Department of Chemical Science and Engineering, School of Materials and Chemical Technology, Tokyo Institute of Technology, Yokohama 226-8502, Japan;; ^b^Earth-Life Science Institute, Tokyo Institute of Technology, Tokyo 152-8550, Japan

**Keywords:** carbonyl sulfide, sulfur isotope, stratospheric sulfate aerosols, gross primary production

## Abstract

Carbonyl sulfide (OCS), the most abundant sulfur-containing gas in the atmosphere, is used as a proxy for photosynthesis rate estimation. However, a large missing source of atmospheric OCS has been inferred. Sulfur isotope measurements (^34^S/^32^S ratio and *δ*^34^S) on OCS are a feasible tool to distinguish OCS sources from oceanic and anthropogenic emissions. Here we present the latitudinal (north–south) observations of OCS concentration and $\delta^{34}$S within Japan. The observed $\delta^{34}$S of OCS of 9.7 to 14.5‰ reflects source and sink effects. Particularly in winter, latitudinal decreases in $\delta^{34}$S values of OCS were found to be correlated with increases in OCS concentrations, resulting an intercept of (4.7 ± 0.8)‰ in the Keeling plot approach. This result implies the transport of anthropogenic OCS emissions from the Asian continent to the western Pacific by the Asian monsoon outflow. The estimated background $\delta^{34}$S of OCS in eastern Asia is consistent with the $\delta^{34}$S of OCS previously reported in Israel and the Canary Islands, suggesting that the background $\delta^{34}$S of OCS in the Northern Hemisphere ranges from 12.0 to 13.5‰. Our constructed sulfur isotopic mass balance of OCS revealed that anthropogenic sources, not merely oceanic sources, account for much of the missing source of atmospheric OCS.

## Significance

Carbonyl sulfide (OCS) is a key proxy of the global photosynthesis rate, but the greatest uncertainty in the OCS cycle is its missing source. Our unique method of measuring sulfur isotope ratios (^34^S/^32^S) of OCS was used in this study to distinguish oceanic and anthropogenic OCS sources. A north–south latitudinal gradient in the ^34^S/^32^S ratio of OCS was found, corresponding to OCS concentrations during wintertime within eastern Asia, providing evidence of the importance of anthropogenic OCS emissions from China. Sulfur isotopic constraints of the atmospheric OCS budget revealed that anthropogenic OCS sources, and not only oceanic OCS sources, are likely to be major constituents of the missing source of atmospheric OCS.

Carbonyl sulfide (OCS or COS, but OCS hereinafter) is the most abundant sulfur-containing gas in the atmosphere, with tropospheric concentrations of approximately 500 ppt (1, 2). Because of its long lifetime (longer than 2 y), OCS can be transported to the stratosphere (3), where it is converted to stratospheric sulfate aerosols, consequently affecting Earth’s radiation balance and stratospheric chemistry (4, 5). Additionally, OCS is consumed by plant leaves together with assimilation of ${CO}_{2}$, but leaves do not emit OCS to the atmosphere by respiration (6). For that reason, OCS is recognized as a proxy for estimating the photosynthesis rate (i.e., gross primary production [GPP]) in ecosystems that have the largest and most uncertain carbon–climate feedback (7). Therefore, better elucidation of the OCS biogeochemical cycle can provide important insights for both radiative forcing and atmosphere–biosphere interaction on the Earth.

A main OCS source is natural oceanic emissions, both as direct OCS emission and indirect OCS emission from oxidations of carbon disulfide (${CS}_{2}$) and dimethylsulfide (DMS) (8). Another main OCS source is indirect emission from anthropogenic ${CS}_{2}$ which originates mainly from rayon production (9) but also from aluminum production, coal combustion, oil refineries, and fuel combustion (10). The main sink of OCS from the atmosphere is uptake by terrestrial vegetation and soil, whereas tropospheric OCS sink reactions (photolysis and reaction with hydroxyl radical or oxygen atom [O(^3^P)]) are minor (8, 11, 12). To date, both top-down and bottom-up approaches have been adopted to investigate the OCS budget, but a recent review (13) pointed out major knowledge gaps in the OCS budget.

The most important point of uncertainty related to the OCS budget is their missing source. Whereas the OCS budget has been regarded as closed in an earlier study reported by Kettle et al. (8), upward revision of the vegetation OCS sink (6) has led to a missing OCS source of 230 to 800 Gg S y^−1^ (14). Berry et al. (14) first proposed the oceanic OCS source to balance the budget residual. This assumption was subsequently supported by top-down approaches observing high atmospheric OCS concentrations ([OCS]) over the Indo-Pacific region (15, 16). By contrast, Lennartz et al. (17) estimated 130 ± 80 Gg S y^−1^ of direct oceanic OCS emission and up to 345 Gg S y^−1^ of total oceanic OCS emission including indirect emission. Based on this result, they inferred that the oceanic OCS emission estimate is too low to account for the missing OCS source. Another candidate for the missing OCS source is anthropogenic origin. A recent global gridded inventory of the primary anthropogenic emission sectors estimates the global anthropogenic OCS source of 406 Gg S y^−1^ (223 to 586 Gg S y^−1^), which roughly corresponds to the estimate of the missing OCS source (10). The lack of observational evidence has led to continuous debate on the issue of whether the missing OCS source is oceanic emission or anthropogenic emission.

One indicator of the atmospheric OCS source might be its sulfur isotopic composition. The isotopic approach assumes distinct *δ*^34^S(OCS) values (*Materials and Methods*) for oceanic OCS (*δ*^34^S = approximately +19‰) and anthropogenic OCS (*δ*^34^S = approximately +3‰) (18, 19). Sulfur isotopic measurement of atmospheric OCS was first developed using a gas chromatograph (GC)-isotope ratio mass spectrometer (IRMS) measuring $S^{+}$ fragmentation ions (20). Using this method, atmospheric $\delta^{34}$S(OCS) values of 4.9‰ were reported from a commercial compressed air sample obtained from one location in Japan (Kawasaki), but this sample from compressed air might have been affected by anthropogenic OCS sources at the sampling site or contamination during compression or preservation processes (21). Furthermore, this method requires hundreds of liters of air per analysis to obtain several nanomoles of OCS. This requirement has limited the applicability of this method. Recently, to overcome this limit, development of a high-volume air sampling system (21) coupled with GC-IRMS method (20) has enabled observation of the atmospheric $\delta^{34}$S(OCS). The value of $\delta^{34}$S(OCS) of (10.5 ± 0.4)‰ was reported for April 2018 at Yokohama, Japan. Almost simultaneously, Angert et al. (19) developed a method using the coupling of a GC with a multicollector-inductively coupled plasma-mass spectrometer, which then enabled the measurement of $\delta^{34}$S(OCS) with a picomole-scale sample.

Based on the similarity of atmospheric $\delta^{34}$S(OCS) at two sites (Israel and the Canary Islands), with values of 12.8 to 13.4‰, Angert et al. (19) reported sulfur isotopic homogeneity of atmospheric OCS. However, differences between $\delta^{34}$S(OCS) of (10.5 ± 0.4)‰ at Yokohama and $\delta^{34}$S(OCS) of (13.2 ± 0.6)‰ obtained in Israel and the Canary Islands indicate that the atmospheric $\delta^{34}$S(OCS) values might not always be constant (21). In addition, several observations have shown variations in [OCS], such as terrestrial seasonality (2) and high level of [OCS] spatially distributed in the boreal summer above the Indo-Pacific region (22) or downwind of air masses from continental China (23). These findings lead us to hypothesize that atmospheric $\delta^{34}$S(OCS) also varies, yielding potentially valuable information about its sources and sinks.

Here we present observations of north–south latitudinal difference of $\delta^{34}$S(OCS) from three sites in Japan: Miyakojima (24°80′N, 125^°^27′E), Yokohama (35^°^51′N, 139°48′E), and Otaru (43^°^14′N, 141°16′E). For the eastern Asian region, synoptic winds are known to be stronger, with westerly circulation (from continental Asia to the Pacific) during winter (January to March). By contrast, winds weaken by May to June; the wind direction changes to southeasterly and continues through August to September (24). In addition, the high [OCS] for the western tropical Pacific near continental China was suggested by both top-down (15, 16) and bottom-up approaches (10). Consequently, the spatial variations of [OCS] and $\delta^{34}$S(OCS) in that region provide an important insight into the applicability of *δ*^34^S for the atmospheric OCS budget constraints.

## Results and Discussion

### [OCS] and $\delta^{34}$S(OCS) for Three Japanese Sites.

**Fig. 1.**
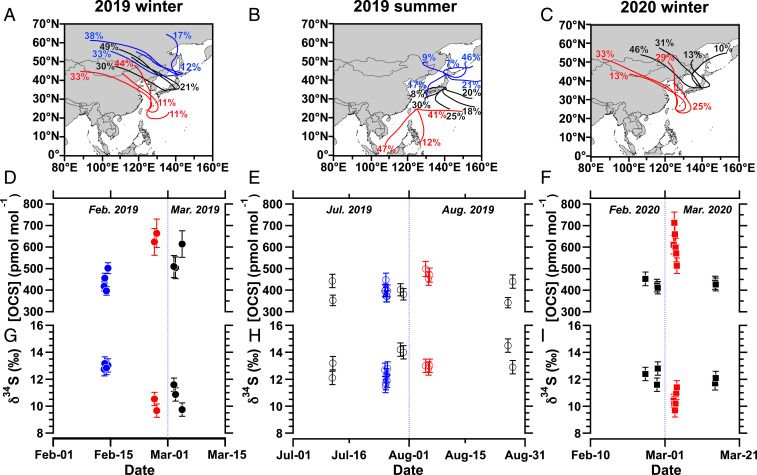
Back trajectory analysis for three Japanese sites for (*A*) 2019 winter, (*B*) 2019 summer, and (*C*) 2020 winter. The time series of (*D*–*F*) [OCS] and (*G*–*I*) $\delta^{34}$S(OCS) during this study period. Red lines and plots represent data for Miyakojima. Black lines and plots represent data for Yokohama. Blue lines and plots represent data for Otaru.

We conducted three campaigns for both wintertime and summertime during 2019 to 2020 (*Materials and Methods*) to evaluate the latitudinal (north–south) difference of [OCS] and $\delta^{34}$S(OCS) within Japan. We investigated the 5-d backward trajectory based on the National Oceanic and Atmospheric Administration (NOAA) Hybrid Single-Particle Lagrangian Integrated Trajectory (HYSPLIT) analysis (25, 26) for the three sampling sites of Miyakojima, Yokohama, and Otaru during the sampling periods. In winter, the history of the air sampled at three sites showed that it mainly originated from the west including continental Asia (Fig. 1 *A* and *C*). The history of the air sampled in Miyakojima was influenced mainly by Chinese megacities including Beijing and Shanghai, whereas Yokohama and Otaru only brushed against the edge of northern China (Fig. 1 *A* and *C*). In summer, however, the air collected at three sites comes from southwest to southeast to the sampling sites (Fig. 1*B*). These features are consistent with typical seasonal wind patterns in East Asia reported elsewhere (24).

The average ±1 SDs for the observed [OCS] for 2019 wintertime were (644 ± 29), (543 ± 62), and (443 ± 46) pmol mol^−1^ for Miyakojima, Yokohama, and Otaru (Fig. 1*D*), respectively, showing north–south latitudinal differences. Similarly, the observed [OCS] for 2020 wintertime for Miyakojima was higher [(611 ± 69) pmol mol^−1^] than for Yokohama [(430 ± 15) pmol mol^−1^] (Fig. 1*F*). In contrast to that for winter, the range of [OCS] for 2019 summertime was narrower (Fig. 1*E*), although the [OCS] in Miyakojima was slightly higher. The high [OCS] observed in Miyakojima was consistent with the observation of [OCS] in Changjiang Estuary located in eastern China, with high [OCS] of 459 to 777 pmol mol^−1^ in wintertime and 417 to 644 pmol mol^−1^ in summer (23). The range of [OCS] in Otaru and Yokohama showed good agreement with earlier observations made at the same latitude (2), which also exhibited a seasonal pattern of high [OCS] in winter and low [OCS] in summer.

The average ±1 SDs for *δ*^34^S(OCS) for 2019 wintertime were (10.1 ± 0.6)‰, (10.7 ± 0.9)‰, and (12.9 ± 0.2)‰ for Miyakojima, Yokohama, and Otaru, respectively (Fig. 1*G*). Similarly, the lower *δ*^34^S(OCS) for Miyakojima compared to that for Yokohama was reproduced in 2020 wintertime (Fig. 1*I*). In summer, *δ*^34^S(OCS) for three sites did not show the clear north–south gradient as observed in winter (Fig. 1*H*). Summarizing the [OCS] and *δ*^34^S(OCS), we have provided additional datasets collected in different seasons from three Japanese sites having different atmospheric origins. The *δ*^34^S(OCS) varied from 9.7 to 14.5‰ and showed a large variation particularly during winter, which provides insight into the OCS source information as we discuss in the following sections.

### OCS Isotopic Composition Reflected by Its Sources and Sinks.

**Fig. 2.**
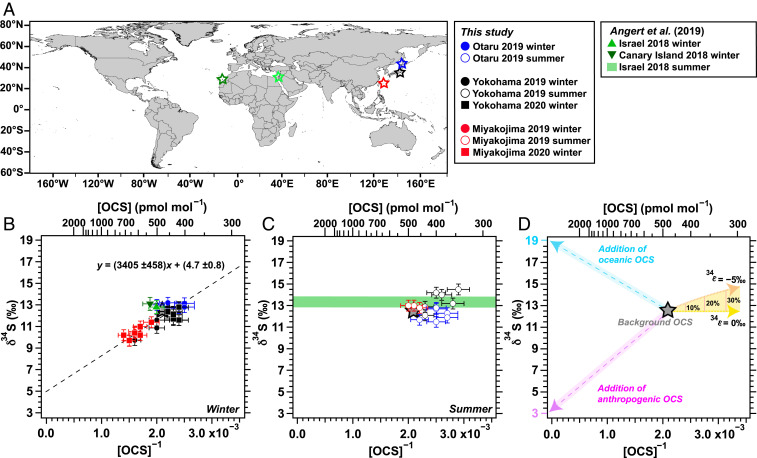
Relation between [OCS] and *δ*^34^S(OCS). (A) Location map and a legend of the sampling sites. (B) Winter. (C) Summer. Error bars represent the relative SD (RSD) of [OCS] (±7%) and SD of *δ*^34^S(OCS) (±0.5‰), and dotted line for B is drawn using York plot (27) with y = 3,405 (±458)x + 4.7 (±0.8). (D) Schematic model of OCS isotopic composition reflecting its source and sink. The gray star denotes the background OCS when assumed that the background OCS possesses [OCS] = 476 pmol mol^−1^ with $\delta^{34}$S(OCS) = 12.5‰. The blue and purple dashed lines with shaded area represent additions of oceanic OCS and anthropogenic OCS, respectively. The dotted yellow and orange curves represent changes in [OCS] and *δ*^34^S(OCS) via the sulfur isotopic fractionation of OCS sink ranging from ^34^*ϵ* = 0 to ^34^*ϵ* = −5‰.

To elucidate the source of additional OCS to the background OCS in the eastern Asian region, we conducted a Keeling plot analysis (*Materials and Methods*) as presented in Fig. 2 *B* and *C*. Fig. 2*D* presents a summary of the scheme showing possible changes in [OCS] and *δ*^34^S from their background values. In this scheme, the two dashed lines that induce increases of [OCS] represent the contribution of sources of OCS from either oceanic or anthropogenic activities processing high or low *δ*^34^S values. In addition, the [OCS] and *δ*^34^S(OCS) can be changed because of isotopic fractionations by several sinks reactions (*Materials and Methods*). We consider three OCS sinks and their sulfur isotopic fractionation constants ^34^*ϵ*: 1) plant uptake [^34^*ϵ* = −5‰ (19)], 2) decomposition by soil microorganisms [^34^*ϵ* = −4‰ to −2‰ (28)], and 3) reaction with OH radical [^34^*ϵ* for 0 to −5‰ at altitudes of 0 to 20 km (29)]. In Fig. 2*D*, we assumed that the background OCS possesses [OCS] = 476 pmol mol^−1^ [annual mean in the Northern Hemisphere (2)] with $\delta^{34}$S = 12.5‰.

In winter, a linear relation between $\delta^{34}$S(OCS) and [OCS]_obs._^−1^ within three Japanese sites was observed with high correlation, as indicated by the coefficient of determination: *R*^2^ = 0.74 (Fig. 2*B*). This correlation indicates that the atmospheric OCS in that season was on the two sources mixing between the background and additional OCS sources. Given that Otaru in the wintertime is not likely to be influenced by direct anthropogenic or oceanic OCS sources, winter $\delta^{34}$S(OCS) values in Otaru [(12.9 ± 0.2)‰] are assumed to represent the background values in East Asia (Fig. 2*B*).

The intercept of the linear regression, which was estimated as $\delta^{34}$S(OCS) of the additional OCS, was low, (4.7 ± 0.8)‰, which is roughly matched with the $\delta^{34}$S of anthropogenic OCS expected to have low $\delta^{34}$S (=approximately +3‰) (18, 19). This result constitutes observational evidence showing that the contribution of anthropogenic OCS caused the north–south [OCS] gradients in eastern Asia in winter. The huge amounts of anthropogenic OCS contributed by Chinese industry (10) and transportation of land air masses from the Asian continent to southern Japan (Fig. 1 *A* and *C*) support this interpretation. It is noteworthy that markedly reduced anthropogenic activities during the coronavirus disease 2019 (COVID-19) outbreak can be expected for 2020 wintertime. However, chemical fiber production in China, including rayon production, which is the largest anthropogenic OCS source through the emission of precursor ${CS}_{2}$ (9, 10), dropped only 33% in January and February 2020 but recovered rapidly after March 2020 to the same level (87%) as that of the same period in 2019 (30). Therefore, we keep the viewpoint that anthropogenic OCS emission from China was still active during the winter of 2019 to 2020.

OCS emission from biomass burning, on the other hand, is not likely to explain this high [OCS] with low $\delta^{34}$S(OCS) observed in southern Japan during winter, given that OCS emission from biomass burning is less important for OCS budget compared to the anthropogenic OCS emission (31). This interpretation is also supported by the fact that OCS emissions from biomass burning are distributed in northern China and Siberia in the study region (31). Hence, if these low *δ*^34^S(OCS) values observed in Miyakojima were due to the biomass burning OCS source, the similar low $\delta^{34}$S(OCS) should have been observed in Otaru where the history of the air is originated from biomass burning active regions such as northern China and Siberia (Fig. 1*A*).

In summer, however, the history of the air sampled at three sites showed that it originated mainly from the south (Fig. 1*B*). In addition, plant uptake, activity of soil microorganisms, and OH radical reactions are active for summer of the Northern Hemisphere. These sulfur isotopic fractionations possibly induce increase of *δ*^34^S with decreased [OCS] (Fig. 2*D*). In that season, no clear latitudinal trend between ${\left[\text{OCS}\right]}_{\text{obs.}}^{-1}$ and *δ*^34^S(OCS) was observed (Fig. 2*C*), contrary to that found in winter. When regarding data for each site, one might be able to hypothesize a complex mixture of both oceanic and anthropogenic OCS sources as well as OCS sink reactions reflected in the variation of [OCS]_obs._^−1^ and $\delta^{34}$S(OCS). For instance, the samples collected at Yokohama during summer showed somewhat high $\delta^{34}$S(OCS) with low [OCS] compared to *δ*^34^S(OCS) and [OCS] for the samples collected at Yokohama in winter (Fig. 2*C*), which might reflect contributions of OCS sinks inducing an increase in $\delta^{34}$S(OCS), as indicated in Fig. 2*D*. For Otaru, the summer $\delta^{34}$S(OCS) values were lower than those for winter, whereas the summer [OCS] in Otaru were higher than those in winter. Considering the history of air sampled at Otaru, which covered Japanese domestic anthropogenic active regions and brushed against the Korean peninsula (Fig. 1*B*), this low $\delta^{34}$S(OCS) found in Otaru might be attributable to anthropogenic OCS possessing lower $\delta^{34}$S. It is particularly interesting that the observations in Miyakojima during the summer showed higher levels of [OCS] and $\delta^{34}$S(OCS) compared to those in other sites (Fig. 2*C*). Given that the history of the air sampled at Miyakojima was mostly from the Indo-Pacific region (Fig. 1*B*) where high levels of [OCS] in the summertime were reported from the top-down approaches (15, 16), one possible explanation for this high [OCS] with high $\delta^{34}$S(OCS) is the contribution of oceanic OCS with high $\delta^{34}$S. As described above, our extended results open up the possibility of sulfur isotopic approach for extracting information about the atmospheric OCS cycle.

### Comparison with Other Sites.

Next, our results are compared with data reported by Angert et al. (19). Based on the similarity of $\delta^{34}$S(OCS) between Israel and the Canary Islands despite the difference in the air mass histories of the two sites, Angert et al. (19) concluded that the *δ*^34^S(OCS) of 12.8 to 13.4‰ is the representative tropospheric background value. Similarly, the *δ*^34^S(OCS) for Otaru during winter, (12.9 ± 0.2)‰, which is assumed to be the background value in East Asia, also shows consistency with the background *δ*^34^S(OCS) reported by Angert et al. (19). This consistency in the background *δ*^34^S(OCS) across a wide area of the Northern Hemisphere suggests that the background *δ*^34^S(OCS) is relatively constant and likely ranges between 12.5 and 13.5‰.

However, it is noteworthy that the relations between [OCS] and *δ*^34^S(OCS) observed in the Canary Islands and Israel in winter were not correlated with those found at the three Japanese sites (Fig. 2*B*). Given the oceanic wind direction to the Canary Islands (19), the slightly high *δ*^34^S(OCS) together with high [OCS] observed at the Canary Islands during winter might be attributable to the contribution from oceanic OCS with high *δ*^34^S. This feature of air mass from oceanic wind direction possessing high *δ*^34^S(OCS) and high [OCS] was observed similarly at Miyakojima in summer, as discussed earlier (Fig. 2*C*). It is particularly interesting that the datasets for Canary Island and Israel in winter and for Miyakojima in summer are almost on the line of mixing between the presumed background OCS and the addition of oceanic OCS (*δ*^34^S = 19‰) in the Keeling plot (Fig. 2 *B* and *C*). The intersection of two mixing lines exhibits *δ*^34^S(OCS) of 12.0 to 12.5‰ (Fig. 2 *B* and *D*), implying that the actual background *δ*^34^S(OCS) might be a bit lower than the estimated *δ*^34^S(OCS) of 12.5 to 13.5‰ described above. Given that an increase in *δ*^34^S(OCS) could be induced by OCS sink reactions (Fig. 2*D* and *Materials and Methods*), this somewhat low estimate of the background *δ*^34^S(OCS) of 12.0 to 12.5‰ might be the result of the exclusion of the isotopic effect by OCS sinks. Presently, it is not easy to judge whether 12.0 to 12.5‰ or 12.5 to 13.5‰ is a more representative background *δ*^34^S(OCS) value. In the following section, we use *δ*^34^S(OCS) within the range of 12.0 to 13.5‰ as the background value to constrain the atmospheric OCS budget based on the isotopic mass balance approach.

### Sulfur Isotopic Constraints for the Atmospheric OCS Budget.

Our results have demonstrated that the *δ*^34^S of atmospheric OCS reflects the contributions of anthropogenic and oceanic OCS emissions. Consequently, *δ*^34^S(OCS) is expected to facilitate isotopic mass balance calculation to estimate the relative contributions of OCS sources. The isotopic mass balance approach relies on the assumption of distinct *δ*^34^S values for each source of OCS (termed end members). To calculate *δ*^34^S(OCS), we assume three end members of oceanic OCS, anthropogenic OCS, and biomass burning OCS. We define *δ*^34^S = +3‰ for anthropogenic OCS, and *δ*^34^S = +19‰ for oceanic OCS. As for OCS from biomass burning, we use *δ*^34^S = +3‰ given that this value was suggested for plant sulfur in an earlier report (18). Using these end members coupled with current OCS budget estimates, we enter the ongoing debate of the missing source of atmospheric OCS as explained below.

The global sources in Kettle’s inventory (8) were 39 Gg S $y^{-1}$ for direct oceanic OCS emission, 237 Gg S $y^{-1}$ for indirect oceanic OCS emission, and 180 Gg S $y^{-1}$ for anthropogenic OCS (Table 1). Although this estimate was balanced with the earlier inventory of OCS sinks, upward revision of vegetation OCS sinks suggests a missing source in the atmospheric OCS budget (6). To balance this, Berry et al. (14) hypothesized 136 Gg S $y^{-1}$ from biomass burning and 600 Gg S $y^{-1}$ of oceanic OCS. Zumkehr et al. (10) estimated anthropogenic OCS emissions of 406 Gg S $y^{-1}$ in the baseline, ranging from 223 to 586 Gg S $y^{-1}$. Recently, OCS emissions from biomass burning are revised and estimated to 60 ± 37 Gg S y^−1^, and total OCS emissions from open burning are insufficient to account for this missing OCS source (31). Therefore, we test two scenarios. One scenario is the oceanic enhancement scenario based on the inventory of Berry et al. (14), which is 600 Gg S y^−1^ of the additional oceanic OCS in addition to Kettle’s inventory (8). Another scenario is the anthropogenic enhancement scenario considering Kettle’s inventory (8) with updated OCS emissions from anthropogenic sources ranging from 223 to 586 Gg S y^−1^ (10) and from biomass burning with 60 Gg S y^−1^ (31). In the anthropogenic enhancement scenario, we also added additional oceanic OCS emission ranging from 270 to 633 Gg S y^−1^ to make an equivalent of OCS sources with the oceanic enhancement scenario (Table 1).

**Table 1.** Estimated global sources and sinks for atmospheric OCS and estimated $\delta^{34}$S(OCS) values

		Scenarios considering the missing OCS source			
			Anthropogenic enhancement		
OCS source	Kettle et al. (8)	Oceanic enhancement	Average	Max.	Min.
Ocean (direct)	39	39	39	39	39
Ocean (indirect)	237	237	237	237	237
Additional oceanic flux	—	600*	450	270	633
Anthropogenic	180	180	406^†^	586^†^	223^†^
Biomass burning	11	136*	60^‡^	60^‡^	60^‡^
$\delta^{34}$S(OCS) (‰)	12.5	14.8	12.7	10.3	15.2

Unit for OCS is Gg S y^−1^.

* Estimated by Berry et al. (14).

^†^ Estimated by Zumkehr et al. (10).

^‡^ Estimated by Stinecipher et al. (31).

When assuming a simplified isotopic mass balance of atmospheric OCS, and neglecting OCS sinks, we obtain

$$\delta^{34}{\text{S}}_{\text{total}}=F_{\text{ocean}}\delta^{34}{\text{S}}_{\text{ocean}}+F_{\text{anth.}}\delta^{34}{\text{S}}_{\text{anth.}}+F_{\text{bb}}\delta^{34}{\text{S}}_{\text{bb}},$$

where $\delta^{34}S_{\text{total}}$, $\delta^{34}S_{\text{ocean}}$, $\delta^{34}S_{\text{anth.}}$, and $\delta^{34}S_{\text{bb}}$ represent $\delta^{34}$S of total and end members of oceanic OCS, anthropogenic OCS, and biomass burning OCS, respectively. Also, $F_{\text{ocean}}$, $F_{\text{anth.}}$, and $F_{\text{bb}}$ denote the fractional contributions of oceanic OCS, anthropogenic OCS, and biomass burning OCS, respectively, and $F_{\text{ocean}}$ + $F_{\text{anth.}}$ + $F_{\text{bb}}$ = 1.

The $\delta^{34}$S(OCS) calculated for the oceanic enhancement scenario yields the high value of 14.8‰ (Table 1), which is too high to reconcile with the estimated background $\delta^{34}$S(OCS) of 12.0 to 13.5‰. On the other hand, the anthropogenic enhancement scenario yields a $\delta^{34}$S(OCS) of 12.7‰ in the baseline (lower estimate, 10.3‰; upper estimate, 15.2‰) (Table 1), including the estimated background $\delta^{34}$S(OCS). Because of this consistency, the isotopic mass balance approach considering the anthropogenic enhancement scenario shows a strong agreement with the proposal in an earlier report (10): anthropogenic sources must be considered as a component of the missing source, along with oceanic OCS emission. When defining the $\delta^{34}$S(OCS) of 12.0 to 13.5‰ as the representative background value, the revised anthropogenic OCS is 350 to 462 Gg S $y^{-1}$. These calculations will be performed more exactly when the sulfur isotopic characterizations of OCS sources and sinks have been completed. Additionally, such observations will be analyzed using a chemical transport model tagged with different OCS sources and considerations of OCS sinks with different isotopic fractionations.

## Conclusions

We demonstrated variation of $\delta^{34}$S(OCS) of 9.7 to 14.5‰ in eastern Asia. Most notably, the north–south latitudinal gradient of $\delta^{34}$S(OCS) during winter is evidence of a very substantial emission of OCS from the Asian continent that is transported to the Western Pacific by outflow from the Asian monsoon. Based on the constant background $\delta^{34}$S(OCS) widely observed in the Northern Hemisphere and the intersection of two mixing lines between background OCS and end members of either anthropogenic or oceanic OCS in the Keeling plot, we estimated that a background $\delta^{34}$S(OCS) in the range from 12.0 to 13.5‰ can be applied to the sulfur isotopic mass balance analysis. The constructed sulfur isotopic mass balance revealed that anthropogenic OCS emission of 350 to 462 Gg S $y^{-1}$ and oceanic enhancement with 394 to 506 Gg S $y^{-1}$ are likely to explain the range of background $\delta^{34}$S(OCS), bridging the knowledge gap of the atmospheric OCS budget.

This sulfur isotopic constraint on atmospheric OCS is an important step, but more observations, together with isotopic characterizations and analysis using a chemical transport model, will enable detailed quantitative conclusions. The increased importance of anthropogenic OCS emission at midlow latitudes has important implications for anthropogenic climate change and stratospheric chemistry in the past and futures. Additionally, given that the historical estimation of GPP is sensitive to the estimate of the anthropogenic OCS inventory (9), a detailed update of the OCS budget constrained by sulfur isotopic approach will enable precise estimation of global historical GPP changes and their interactions with global change.

## Materials and Methods

### Sampling and Measurement.

OCS sampling was conducted for three campaigns: 1) February to March 2019 at three Japanese sites, Miyakojima (24°80′N, 125°27′E), Yokohama (35°51′N, 139°48′E), and Otaru (43°14′N, 141°16′E); 2) July to August 2020 at three sites; and 3) February to March 2020 at Miyakojima and Yokohama. The sampling details are summarized in *SI Appendix*, Tables S1–S3. The buildings used at Miyakojima and Otaru face the ocean. They are more than 5 km distant from possible local sources. Sampling at Yokohama was performed at the Suzukakedai campus of the Tokyo Institute of Technology, located in an urban site and near a highway. OCS was collected at more than 10 m above ground level.

Sampling and the corresponding measurements applied a method developed in our earlier works (20, 21). Briefly, the system consisted of two parts: a large volume air sampling system and an online OCS purification system. OCS in air was collected in a sampling tube at temperatures of −140 to −110°C by vapor of the liquid $N_{2}$ in a dewar with a low-volume diaphragm pump flow of (5.0 ± 0.25) L min^−1^. The OCS in the sampling tube were transferred to the adsorption tube cooled at −72°C using dry ice and ethanol. The adsorption tubes were stored at −72°C in dry ice or in a freezer until analysis. The adsorption tube containing OCS sample was connected to the purification system. The purified OCS was injected to an isotope ratio mass spectrometer (MAT253; Thermo Fisher Scientific) to ascertain the OCS amount and $\delta^{34}$S(OCS). Uncertainties of the [OCS] and $\delta^{34}$S(OCS) values were ±7% and ±0.5‰, respectively (21).

### Back-Trajectory Analysis.

Five-day backward trajectories were calculated for February to March 2019, July to August 2019, and February to March 2020 using the HYSPLIT model (25, 26), to obtain general seasonal characteristics of air mass origin for each region. The trajectories were started at 0:00 and 12:00 UTC each day at half the height of the planetary boundary, and the trajectories that contacted the surface (0 m height) were rejected. The calculated air mass trajectories were clustered. Then the proportions of transportation paths were calculated for each sampling site using TrajStat software (32).

### Definitions.

Sulfur isotopic composition is defined from the following equation as

δS34=(S34/S32)sample/(S34/S32)standard −1, [1]

which is described in a per mil (‰) notation relative to the international standard Vienna Canyon Diablo Troilite.

The Keeling plot analysis (33) to our results is expressed as

$$\delta^{34}{\text{S}}_{\text{obs.}}={\left[\text{OCS}\right]}_{\text{bg.}}\left(\delta^{34}{\text{S}}_{\text{bg.}}-\delta^{34}{\text{S}}_{\text{s}}\right)\left(1/{\left[\text{OCS}\right]}_{\text{obs.}}\right)+\delta^{34}{\text{S}}_{\text{s}},$$ [2]

where Sobs.34, Sbg.34, Ss.34, ${\left[\text{OCS}\right]}_{\text{obs.}}$, and ${\left[\text{OCS}\right]}_{\text{bg.}}$ represent the observed $\delta^{34}$S(OCS), $\delta^{34}$S of the background OCS, the $\delta^{34}$S of the additional OCS, the observed [OCS], and the background [OCS], respectively.

When OCS is consumed by the sink reaction, the reaction induces isotopic enrichment or depletion in a reservoir species. This isotopic fractionation is commonly quantified using the isotopic fractionation constant ^34^ϵ,

$${}^{34}\epsilon \;={\;}^{34}k{/}^{32}k-1,$$ [3]

where ^32^*k* and ^34^*k* represent the rate constants associated with the OCS sink reaction. When defining ^34^*ϵ* = −5‰ for OCS sink in combination with spring to fall [OCS] difference of approximately 30% (2) in a closed system, the estimate ^34^S enrichment yields 1.8‰ in *δ*^34^S (Fig. 2*D*).

## Supplementary Material

Supplementary File

## Data Availability

All data are presented in *SI Appendix*, Tables S1–S3.
