# Supplementary material for: Constraining the atmospheric OCS budget from sulfur isotopes
Source: Proc Natl Acad Sci U S A. 2020 Aug 5;117(34):20447–52. doi: 10.1073/pnas.2007260117 (PMC7456067; doi:10.1073/pnas.2007260117)
Supplement: Supplementary File [file pnas.2007260117.sapp.pdf]

PNAS
www.pnas.org

Supplementary Information for

Constraining the atmospheric OCS budget from sulfur isotopes

Shohei Hattori, Kazuki Kamezaki and Naohiro Yoshida

Corresponding authors: Shohei Hattori and Kazuki Kamezaki
E-mail: hattori.s.ab@m.titech.ac.jp (SH), kame3974@gmail.com (KK)

This PDF file includes:

Tables S1 to S3

www.pnas.org/cgi/doi/10.1073/pnas.2007260117

Table S1. The information of OCS sampling and measurement in winter 2019

Sample ID Site-Number	Site	Starting time JST	Day of the week	Sampling time min	Integrated air volume L	OCS amount[a] nmol	[OCS][b] pmol mol^{-1}	1/[OCS] (pmol mol^{-1})$^{-1}$	δ^{34}S(OCS) ‰
O-1	Otaru	Feb. 13 2019 09:50	Wed.	49	245 ±12	4.2 ±0.3	418 ±30	0.0024 ±0.00017	12.7 ±0.5
O-2	Otaru	Feb. 13 2019 13:45	Wed.	30	150 ±8	2.7 ±0.2	456 ±32	0.0022 ±0.00015	13.2 ±0.5
O-3	Otaru	Feb. 13 2019 23:18	Wed.	70	350 ±18	5.7 ±0.3	397 ±28	0.0025 ±0.00018	12.8 ±0.5
O-4	Otaru	Feb. 14 2019 08:00	Thu.	30	150 ±8	3.1 ±0.2	502 ±35	0.0020 ±0.00014	13.0 ±0.5
M-1	Miyakojima	Feb. 25 2019 16:30	Mon.	30	150 ±8	3.8 ±0.2	624 ±44	0.0016 ±0.00011	10.5 ±0.5
M-2	Miyakojima	Feb. 26 2019 05:30	Tue.	34	170 ±9	4.6 ±0.3	664 ±46	0.0015 ±0.00011	9.7 ±0.5
Y-1	Yokohama	Mar. 02 2019 10:30	Sat.	30	150 ±8	3.1 ±0.2	510 ±36	0.0020 ±0.00014	11.6 ±0.5
Y-2	Yokohama	Mar. 02 2019 20:00	Sat.	30	150 ±8	3.1 ±0.2	504 ±35	0.0020 ±0.00014	10.9 ±0.5
Y-3	Yokohama	Mar. 04 2019 10:30	Mon.	30	150 ±8	3.8 ±0.2	614 ±43	0.0016 ±0.00011	9.7 ±0.5

[a] Determined by the peak area of OCS using an IRMS with RSD uncertainty of 7%.
[b] Calculated from OCS amount divided by integrated air volume.

Table S2. The information of OCS sampling and measurement in summer 2019

Sample ID<br>Site-Number	Site	Starting time<br>JST	Day of the week	Sampling time<br>min	Integrated air volume<br>L	OCS amount[a]<br>nmol	[OCS][b]<br>pmol mol^{-1}	1/[OCS]<br>(pmol mol^{-1})$^{-1}$	δ^{34}S(OCS)<br>‰
Y-4	Yokohama	Jul. 11 2019 11:00	Thu.	30	150 ±8	2.7 ±0.2	443 ±31	0.0023 ±0.00016	12.1 ±0.5
Y-5	Yokohama	Jul. 11 2019 14:00	Thu.	30	150 ±8	2.2 ±0.2	353 ±25	0.0028 ±0.00020	13.2 ±0.5
O-5	Otaru	Jul. 25 2019 13:00	Thu.	40	200 ±10	3.2 ±0.2	397 ±28	0.0025 ±0.00018	12.7 ±0.5
O-6	Otaru	Jul. 25 2019 16:00	Thu.	40	200 ±10	3.2 ±0.2	400 ±28	0.0025 ±0.00016	11.5 ±0.5
O-7	Otaru	Jul. 25 2019 19:00	Thu.	40	200 ±10	3.6 ±0.3	448 ±31	0.0022 ±0.00019	11.7 ±0.5
O-8	Otaru	Jul. 25 2019 22:00	Thu.	40	200 ±10	3.0 ±0.2	372 ±26	0.0027 ±0.00019	12.3 ±0.5
O-9	Otaru	Jul. 26 2019 01:00	Fri.	40	200 ±10	3.0 ±0.2	371 ±26	0.0027 ±0.00018	11.9 ±0.5
O-10	Otaru	Jul. 26 2019 05:00	Fri.	40	200 ±10	3.2 ±0.2	397 ±28	0.0025 ±0.00018	12.8 ±0.5
Y-6	Yokohama	Jul. 29 2019 16:00	Mon.	30	150 ±8	2.5 ±0.2	402 ±28	0.0025 ±0.00017	14.2 ±0.5
Y-7	Yokohama	Jul. 30 2019 11:00	Tue.	30	150 ±8	2.3 ±0.2	381 ±27	0.0026 ±0.00018	14.0 ±0.5
M-3	Miyakojima	Aug. 5 2019 09:30	Mon.	40	200 ±10	4.0 ±0.3	499 ±35	0.0020 ±0.00014	13.0 ±0.5
M-4	Miyakojima	Aug. 6 2019 03:30	Tue.	40	200 ±10	3.7 ±0.3	454 ±32	0.0022 ±0.00015	12.8 ±0.5
M-5	Miyakojima	Aug. 6 2019 11:30	Tue.	40	200 ±10	3.8 ±0.3	470 ±33	0.0021 ±0.00015	13.0 ±0.5
Y-8	Yokohama	Aug. 27 2019 10:00	Tue.	40	200 ±10	2.8 ±0.2	342 ±24	0.0029 ±0.00020	14.5 ±0.5
Y-9	Yokohama	Aug. 28 2019 16:50	Wed.	40	200 ±10	3.5 ±0.2	440 ±31	0.0023 ±0.00016	12.9 ±0.5

[a] Determined by the peak area of OCS using an IRMS with RSD uncertainty of 7%.
[b] Calculated from OCS amount divided by integrated air volume.

Table S3. The information of OCS sampling and measurement in winter 2020

Sample ID<br>Site-Number	Site	Starting time<br>JST	Day of the week	Sampling time<br>min	Integrated air volume<br>L	OCS amount[a]<br>nmol	[OCS][b]<br>pmol mol^{-1}	1/[OCS]<br>(pmol $mol^{-1})^{-1}$	δ^{34}S(OCS)<br>‰
Y-10	Yokohama	Feb. 24 2020 17:30	Mon.	40	200 ±10	3.6 ±0.3	453 ±32	0.0022 ±0.00015	12.4 ±0.5
Y-11	Yokohama	Feb. 27 2020 20:00	Thu.	40	200 ±10	3.4 ±0.2	422 ±30	0.0024 ±0.00017	11.6 ±0.5
Y-12	Yokohama	Feb. 28 2020 01:00	Fri.	40	200 ±10	3.3 ±0.2	412 ±29	0.0024 ±0.00017	12.8 ±0.5
M-6	Miyakojima	Mar. 3 2020 08:00	Tue.	30	150 ±8	3.7 ±0.3	611 ±43	0.0016 ±0.00011	10.4 ±0.5
M-7	Miyakojima	Mar. 3 2020 12:00	Tue.	40	200 ±10	5.7 ±0.4	713 ±50	0.0014 ±0.00010	10.2 ±0.5
M-8	Miyakojima	Mar. 3 2020 16:00	Tue.	40	200 ±10	5.3 ±0.4	660 ±46	0.0015 ±0.00011	9.7 ±0.5
M-9	Miyakojima	Mar. 3 2020 20:00	Tue.	40	200 ±10	4.8 ±0.3	599 ±42	0.0017 ±0.00012	10.2 ±0.5
M-10	Miyakojima	Mar. 4 2020 00:00	Wed.	40	200 ±10	4.6 ±0.3	572 ±40	0.0017 ±0.00012	11.0 ±0.5
M-11	Miyakojima	Mar. 4 2020 04:00	Wed.	40	200 ±10	4.1 ±0.3	514 ±36	0.0019 ±0.00014	11.4 ±0.5
Y-13	Yokohama	Mar. 14 2020 11:00	Sat.	40	200 ±10	3.5 ±0.2	435 ±30	0.0023 ±0.00016	11.7 ±0.5
Y-14	Yokohama	Mar. 14 2020 15:00	Sat.	40	200 ±10	3.4 ±0.2	427 ±30	0.0023 ±0.00016	12.1 ±0.5

[a] Determined by the peak area of OCS using an IRMS with RSD uncertainty of 7%.
[b] Calculated from OCS amount divided by integrated air volume.
